# Healthcare workers' attitudes toward and factors influencing their acceptance of an annual COVID-19 booster vaccine: a cross-sectional study in Palestine

**DOI:** 10.1186/s12913-024-11016-w

**Published:** 2024-05-14

**Authors:** Beesan Maraqa, Zaher Nazzal, Hassan Baroud, Mahmoud Douden, Yousef El Hamshary, Tala Jalamneh

**Affiliations:** 1 Ministry of Health, Ramallah, Palestine; 2 Department of Medicine, Faculty of Medicine and Health Sciences, An-Najah National University, Nablus, Palestine; 3 Department of Family Medicine, Palestinian Medical Council, Gaza, Palestine; 4 Community and Family medicine department, College of Medicine, Hebron University, Hebron, Palestine; 5 Department of Internal Medicine, An-Najah National University Hospital, Nablus, Palestine

**Keywords:** COVID-19, Healthcare workers, Annual booster vaccine, Acceptance, Hesitancy

## Abstract

**Background:**

The emergence of several SARS-CoV-2 variants may necessitate an annual COVID-19 booster vaccine. This study aimed to evaluate healthcare workers' (HCWs) acceptance of a COVID-19 yearly booster vaccine if recommended and its association with their attitudes and burnout levels.

**Methods:**

We used an online self-administered questionnaire to conduct a cross-sectional study of all HCWs in the West Bank and Gaza Strip of Palestine between August and September 2022. We used the Vaccination Attitudes Examination scale to assess HCWs' vaccination attitudes and the Maslach Burnout Inventory to assess work-related Burnout. In addition, we conducted logistic regression to identify factors independently associated with the acceptance of the booster vaccine.

**Results:**

The study included 919 HCWs; 52.4% were male, 46.5% were physicians, 30.0% were nurses, and 63.1% worked in hospitals. One-third of HCWs (95% CI: 30.5%-36.7%) said they would accept an annual COVID-19 booster vaccine if recommended. HCWs who are suspicious of vaccine benefits [aOR = .70; 95%CI: .65-.75] and those concerned about unforeseeable future effects [aOR = .90; 95%CI: .84-.95] are less likely to accept the booster vaccine if recommended, whereas those who receive annual influenza vaccine are more likely to get it [aOR = 2.9; 95%CI: 1.7–5.0].

**Conclusion:**

Only about a third of HCWs would agree to receive an annual COVID-19 booster vaccine if recommended. Mistrust of the vaccine's efficacy and concerns about side effects continue to drive COVID-19 vaccine reluctance. Health officials need to address HCWs' concerns to increase their acceptance of the annual vaccine if it is to be recommended.

**Supplementary Information:**

The online version contains supplementary material available at 10.1186/s12913-024-11016-w.

## Introduction

COVID-19 first appeared in Wuhan, China, in December 2019 and rapidly spread worldwide, prompting the World Health Organization (WHO) to declare a pandemic in March 2020. As of September 2022, it is estimated that 610 million people have contracted the disease, with 1.5 million fatalities [1]. In Palestine, approximately 620,000 cases have been confirmed, resulting in 5,403 deaths over the same period [2]. It also significantly impacted the healthcare system, increasing admissions and infection of healthcare workers (HCWs) and decreasing essential healthcare utilization [3].

Vaccination is among the most advantageous health interventions due to its positive effects on population health and the economy. COVID-19 vaccination has effectively prevented the disease and lowered the risk of hospitalization and death [4]. This protection, however, declines over time due to waning immunity and, most importantly, the emergence of new virus variants [5]. Several SARS-CoV-2 variants have emerged since the pandemic's beginning, the most significant of which were Alpha, Beta, Gamma, Delta, and Omicron. These variants were linked to increased transmissibility or virulence and decreased vaccination effectiveness and were responsible for multiple waves of infections worldwide [6]. The World Health Organization mentions these to explain why we may need COVID-19 booster doses [7] and raises the possibility that a COVID-19 booster vaccine is required. Some wealthy countries are taking steps in this direction by promoting annual COVID-19 vaccination. According to the White House, the United States could have a COVID-19 booster schedule similar to the annual influenza vaccine [8]. In addition, yearly COVID-19 vaccinations are expected in the United Kingdom, particularly for HCWs, to protect against anticipated COVID-19 surges during the winter [9].

Primary prevention strategies rely on the vaccination practices and attitudes of HCWs. Positive attitudes towards vaccination protect themselves, their families, and patients and encourage others to adopt it. They serve as vaccine enablers and communicators to patients and the public [10], but their reluctance to accept booster doses may undermine vaccine trust [11]. Several studies have shown that HCW vaccination hesitancy is variable. A rapid systematic review found that vaccine acceptance varied widely, ranging from 27.7% to 77.3% [12]. Another meta-analysis study revealed that HCWs' COVID-19 vaccine acceptance pooled effect value was 51% [13], while it was generally low in Africa [14].

Similarly, studies conducted in Palestine shortly before the vaccine’s initial launch showed that HCWs hesitated to accept the vaccination [15, 16]. Hesitancy has been linked to various factors, including sex, profession, education, previous influenza vaccination, self-perceived risk, vaccine safety and effectiveness concerns, and many other factors [12–14]. A recently published study reported that almost a quarter of British HCWs hesitated to receive a regular COVID-19 vaccination. Age, ethnicity, previous COVID-19 vaccination, attitudes, and influenza vaccinations in previous seasons were all associated with regular COVID-19 vaccination [17].

Negative attitudes toward healthy behaviors may result from the stress of HCWs [18]. Burnout, a psychological work-related stress syndrome that develops in response to occupational stressors [19], is common among HCWs, exacerbated by the COVID-19 pandemic [20]. It consists of three elements: emotional exhaustion (EE), depersonalization (DP) (becoming emotionally distant or indifferent), and a diminished sense of personal accomplishment (PA) [19]. Burnout has many consequences, including decreased job satisfaction, absenteeism, anxiety, depression, substance abuse, suboptimal patient care, and impaired quality of care [21].

Palestinian HCWs experience high levels of Burnout, which can be attributed to the pressures of daily work and the challenges Palestine faces as a developing country still under occupation [22, 23]. In addition to its direct effects, Burnout can have indirect consequences on HCWs, leading to a decline in service quality characterized by reduced adherence to guidelines, ineffective communication, compromised patient outcomes and diminished safety standards [24].

This is the first study in the Eastern Mediterranean Region to examine HCWs' willingness to accept and attitudes toward an annual COVID-19 booster vaccine. A better understanding of the acceptance rate of an annual COVID-19 booster vaccine among HCWs and the factors influencing it would aid in developing interventions to reduce hesitancy and increase uptake. The primary objective of this study is to assess the willingness of healthcare workers (HCWs) to accept an annual COVID-19 booster vaccine, should it be recommended. Secondary objectives are 1. investigating the relationship between HCWs' attitudes towards the COVID-19 vaccine and their willingness to accept the booster dose, 2. examining the association between HCWs’ burnout levels and their acceptance of the vaccine booster dose. The primary hypotheses to be examined in this study are: 1. There is a significant relationship between HCWs' attitudes towards the COVID-19 vaccine and their acceptance of the vaccine booster dose, and 2. There is a significant association between HCWs' level of Burnout and their acceptance of the COVID-19 vaccine booster dose.

## Methods

### Study design and population

We conducted a cross-sectional study from August to September 2022 using an online self-administered questionnaire. We targeted all Palestinian HCWs, including physicians, nurses, and allied health professionals such as lab technicians, radiology technicians, and occupational and physical therapists. These HCWs worked in various healthcare settings throughout the West Bank and Gaza Strip, including hospitals and primary healthcare clinics run by government and non-governmental organizations. The inclusion criteria were currently HCW, including physicians, nurses, and paramedics, and working in a health care setting. The inclusion of this heterogeneous group of HCWs (physicians, nurses, etc.) with diverse background characteristics is because they all share a high susceptibility to the infection. More importantly, they all share a characteristic of still being seen as a trustworthy information source, promoting vaccination in low-resource settings and among other groups hesitant to vaccinate [10, 25, 26].

A minimum sample size of 911 HCWs was necessary to determine the prevalence of hesitancy for annual COVID-19 vaccination. It was calculated using the formula n = [DEFF*Np(1-p)]/[(d2/Z21-α/2*(N-1) + p*(1-p), where Z = 1.96 is the confidence level statistic, DEFF = 1 is design effect, P = 33% is the estimated proportion of HCWs willing to receive an annual COVID-19 booster vaccine based on previous studies [15], and d = 3% is the absolute precision. Thus, a minimum sample size of 911 HCWs was necessary to achieve the study objectives.

We employed a convenience sampling technique to recruit participants for our study. Specifically, we distributed Google Forms links and introductory invitations to closed institutional groups of HCWs through messaging platforms such as WhatsApp and Messenger. This method allowed us to efficiently reach out to healthcare professionals within specific institutional networks, ensuring a diverse representation of participants from various healthcare settings. The study was carried out in compliance with current laws on ethical standards and privacy protection. Along with the questionnaire, we enclosed an introductory note explaining the study's purpose and assured respondents that their anonymity and the confidentiality of their responses would be strictly protected. In addition, participants were asked to confirm their agreement with the information provided and their willingness to participate online by tapping the "I agree" item. The Institutional Review Board of An-Najah National University approved the study [Ref. #: Med. August 2022/26].

### Measurement tools

The research team developed the questionnaire for this study using related literature and previous studies (Supplementary File 1). Before being finalized and distributed to participants, the questionnaire was reviewed by three experts in the field and piloted with 30 HCWs. It is divided into four sections. The first section assessed HCWs' background, professional, and clinical characteristics, which included age, gender, profession, workplace place, marital status, smoking status, and presence of chronic diseases. The second section evaluated variables associated with COVID-19 in terms of the history of COVID-19 by polymerase chain reaction (PCR), history of vaccination, and vaccine side effects.

Furthermore, we assessed the annual influenza vaccine uptake by asking, "Do you get the influenza vaccine yearly?". The questionnaire incorporated a specific question to evaluate the primary outcome of the study, directly inquiring of HCWs whether they would agree to receive an annual COVID-19 booster vaccine if recommended, allowing for three potential responses: "Yes," "No," or "Not decided.” Participants were subsequently categorized as acceptant or hesitant, with acceptant referring to HCWs who responded yes. In contrast, hesitant encompassed those who replied negatively or expressed uncertainty by stating "No" or "Not decided."

The third section used the Vaccination Attitudes Examination Scale (VAX) adjusted to the COVID-19 vaccine to assess the HCWs' vaccination attitudes [27]. It has 12 items divided into four sub-scales: mistrust of vaccine benefits, worries over future effects, concerns about commercial profits, and preference for natural immunity. Each subscale has three items scored from 1 (strongly agree) to 6 (strongly disagree), except items of the first subscale, which are reversely coded. Higher scores indicate anti-vaccination attitudes. We used the Arabic version of the VAX scale, which has been used in previous studies and had high internal consistency [28]. The internal consistency coefficient (Cronbach's α) of the VAX scale used in this study was 0.84.

The last section evaluated HCWs' work-related Burnout using the Maslach Burnout Inventory (MBI) [29]. It is a 22-item tool that asks participants, on a 7-point Likert scale (from 0, 'never,' to 6, 'daily'), how frequently they had recently experienced specific feelings related to their work. The MBI is the most commonly used tool, and it consists of three scales: EE (nine items), which measures one's emotional and physical exhaustion as a result of his work; DP (five items), which assesses work-related stress, lack of feeling, impersonal responses to patient care, and reduced empathy; and PA (eight items), which evaluates the individuals' perception of their work and reflects how they perceive its significance. High scores on the EE or DP scales or low scores on the PA scale indicate a high level of Burnout. While no definite cut-off points for MBI subscales exist, we used the following cut-off points from a previous study on HCWs in the region [30]: Burnout was high on EE and PA, and DP when the scores were ≥ 35, ≤ 29, or ≥ 11, respectively. It was moderate on EE, PA, and DP when scores were 21–30, 41–36, and 6–10, respectively. HCWs who ranked high in all three dimensions were considered to have very high Burnout. Internal consistency (Cronbach's) values for the EE, DP, and PA dimensions used in this study were all high: 0.88, 0.80, and 0.90, respectively.

### Data analysis

Data entry and analysis were done with the IBM SPSS Statistics for Windows, version 21 (IBM Corp., Armonk, NY, USA). We summarized categorical variables using frequency distributions and proportions, and the associations were tested using the chi-square test. Next, the Kolmogorov–Smirnov test was used to determine the normality of continuous variables, which revealed that they were normally distributed. The data was then summarized using mean and standard deviation (SD), and the association between different groups was conducted using the independent t-test. The binary logistic regression model accounted for confounders and assessed factors independently associated with vaccine booster dose hesitancy. Adjusted odds ratios (aOR) and 95% confidence intervals (CI) were used to present the findings. We computed the area under the curve-receiver operating characteristic (AUC-ROC) to evaluate the performance of the multivariate logistic regression model using fivefold cross-validation and 100 iterations. The AUC equals 82%, which is considered an excellent capacity to discriminate between an acceptant and a hesitant class. This step was done by R cran version 4.3. The significance level was set at a *P*-value of less than 0.05.

## Results

### Background characteristics

The study included 919 HCWs in total. Table 1 shows the sociodemographic and work-related characteristics of the study sample. It was found that 52.4% of respondents were male, 58.7% were under 30, and 53.5% were married. Almost half of the participants were employed by the government, 46.5% were physicians, 30.0% were nurses, and 63.1% worked in hospitals. About one-fourth of HCWs smoked, and 7.6% had chronic diseases.

**Table 1:** Participants' background and demographic characteristics with the willingness to get an annual COVID booster vaccine (*n* = 919)

*Characteristic*	Total *n (%)*	Annual COVID-19 booster vaccine		*P*-value*
		**Acceptant**	**Hesitant**	
***Sex***				
Male	480 (52.4%)	185 (38.5%)	295 (61.5%)	**.001**
Female	437 (47.6%)	122 (27.9%)	315 (72.1%)	
***Age***				
Under 30 years	539 (58.7%)	168 (34.5%)	353 (65.5%)	.635
30–39 years	263 (28.6%)	82 (31.2%)	181 (68.8%)	
≥ 40 years	114 (12.4%)	39 (34.2%)	75 (65.8%)	
**Marital status**				
Married	492 (53.5%)	155 (31.5%)	337 (68.5%)	.158
Single	423 (46.4%)	153 (35.9%)	273 (64.1%)	
**Profession**				
Physicians	425 (46.5%)	185 (43.5%)	240 (56.5%)	** < .001**
Nurses	274 (30.0%)	73 (26.6%)	201 (73.4%)	
Allied health professionals	215 (23.5%)	49 (22.8%)	166 (77.2%)	
***Health care setting***				
Governmental	467 (51.8%)	170 (35.7%)	306 (64.3%)	.176
Non- Governmental	82 (8.9%)	22 (26.8%)	60 (73.2%)	
Private	343 (37.3%)	107 (31.2%)	236 (68.8%)	
**Work division**				
Hospitals	543 (63.1%)	206 (37.9%)	337 (62.1%)	**.001**
Primary health care	232 (27.0%)	68 (29.3%)	164 (70.7%)	
Others^b^	85 (9.9%)	17 (20.0%)	68 (80.0%)	
**Smoking**				
Non-smoker	648 (60.5%)	213 (32.9%)	435 (67.1%)	.327
ex-smoker	33 (3.6%)	15 (45.5%)	18 (54.5%)	
Smoker	238 (25.9%)	80 (33.5%)	158 (66.4%)	
***Chronic disease***^a^				
Yes	70 (7.6%)	20 (28.6%)	50 (71.4%)	.362
No	849 (92.4%)	288 (33.9%)	561 (66.1%)	

^*^Chi-squared test

^a^Chronic diseases include hypertension, diabetes, cancer, chronic kidney disease, chronic respiratory diseases, and others

^b^Include private clinics, laboratories, pharmacies, etc.

#### Annual COVID-19 booster vaccine

**Fig. 1:**
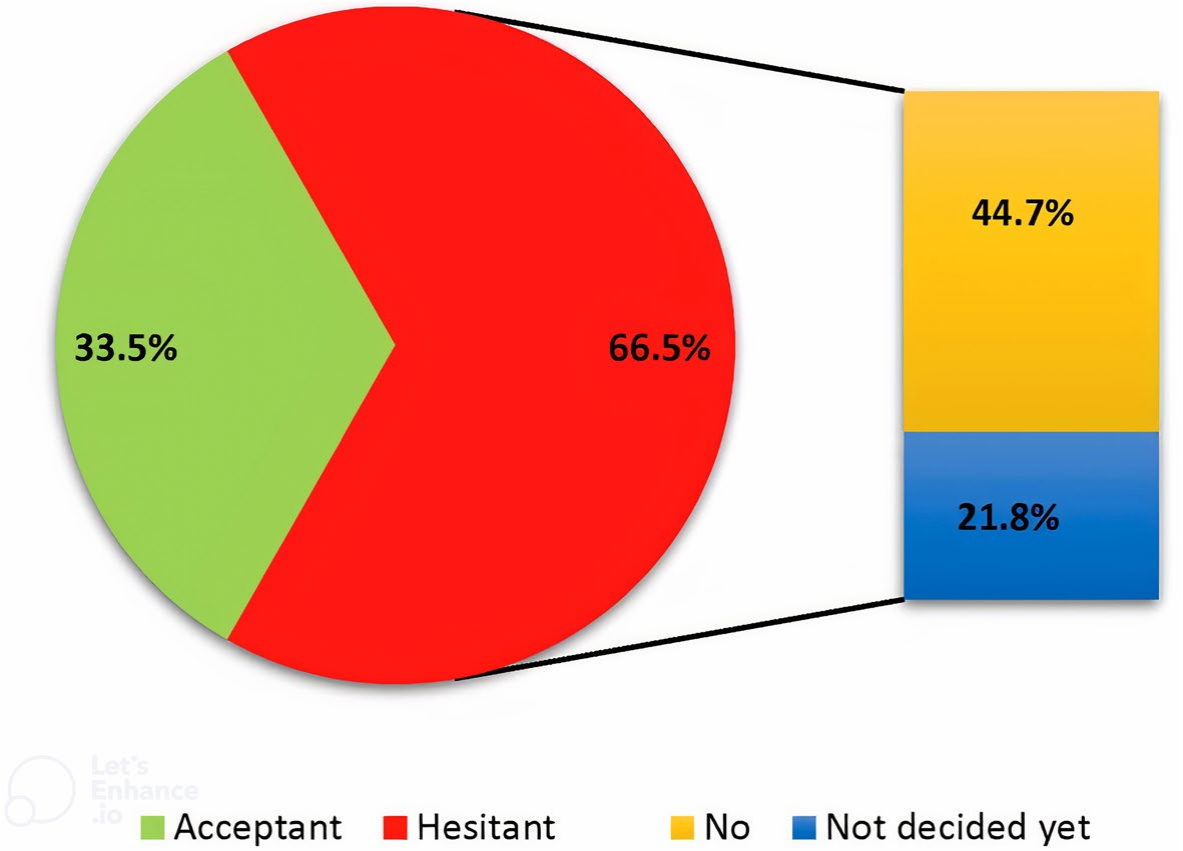
Healthcare workers' responses about receiving an annual COVID-19 booster vaccine

Overall, 308 HCWs (33.5%; 95% CI: 30.5%-36.7%) said they would accept an annual COVID-19 booster vaccine if recommended, while 611 (66.5%; 95% CI: 44.7%- 69.5%) were hesitant; 44.7% said no, and 21.8% were undecided (Fig. 1).

The bivariate analysis revealed significant variations in booster vaccine acceptability based on gender, profession, and work division. For instance, male HCWs were more likely to accept the annual COVID-19 booster vaccine than females (52.4% vs 27.9%; *P* value 0.001). Furthermore, physicians were more likely to accept the annual COVID-19 booster vaccine than nurses (51.8% vs. 30.0%; *P* value < 0.001), and HCWs working in hospitals exhibit higher levels of booster vaccine acceptance than HCWs working at PHC centers (63.1% vs 27.0%; *P* value 0.001) (Table 1). Additionally, HCWs who had previously received the COVID-19 vaccine (52.4% vs. 27.9%; *P* value < 0.001) and those who routinely received an annual influenza vaccine (56.9% vs. 29.9%; *P* value < 0.001) demonstrated a greater likelihood of acceptance (Table 2). On the other hand, HCWs who are suspicious of vaccine benefits and concerned about unforeseeable future consequences are more likely to be hesitant (Table 2).

**Table 2:** HCWs' history of COVID-19 infection, COVID-19 vaccine uptake, and vaccination attitudes with the willingness to get an annual COVID booster vaccine (*n* = 919)

	**Total *n (%)***	**Annual COVID-19 booster vaccine**		*P*-value*
		**Acceptant**	**Hesitant**	
**Previous COVID-19**				
Yes	580 (63.3%)	196 (33.8%)	384 (66.2%)	.631
No	335 (36.6%)	108 (32.2%)	227 (67.8%)	
***Dealt with post-vaccination severe COVID-19 cases***				
Yes	529 (58.7%)	186 (34.5%)	353 (65.5%)	.447
No	380 (41.3%)	122 (32.1%)	258 (67.9%)	
**COVID-19 vaccination**				
Yes	722 (78.6%)	270 (37.4%)	452 (62.6%)	** < .001**
No	197 (21.4%)	38 (19.3%)	159 (80.7%)	
***Side effects of* COVID-19 vaccine**				
Yes	412 (42.9%)	143 (34.7%)	269 (65.3%)	.085
No	310 (57.1%)	127 (41.0%)	183 (59.0%)	
***Received annual influenza vaccine***				
Yes	123 (13.4%)	70 (56.9%)	53 (43.1%)	** < .001**
No	796 (86.6%)	238 (29.9%)	558 (70.1%)	
***Mistrust of vaccine benefits*** (*Mean* ± *SD)*	9.2 ± 3.9	6.6 ± 2.7	10.6 ± 3.8	** < .001**
***Worries over future effects**** (Mean* ± *SD)*	14.2 ± 3.2	13.7 ± 2.8	14.4 ± 3.2	**.002**
***Concerns about commercial profits**** (Mean* ± *SD)*	10.8 ± 4.0	9.5 ± 4.0	11.4 ± 3.9	.242
***Preference for natural immunity*** (*Mean* ± *SD)*	12.4 ± 3.9	11.3 ± 3.8	12.9 ± 3.8	.936

^*^Chi-squared test and *independent t-test*

#### Burnout among HCWs

Overall, 273 HCWs (29.7%) reported high EE, 454 HCWs (49.4%) reported high DP and 202 HCWs (22.0%) reported high reduced PE. In addition, higher levels of vaccine hesitancy were observed among HCWs with moderate and high levels of Burnout in the three domains, but none reached statistical significance (Table 3).

**Table 3:** Burnout among HCWs and its association with the willingness to get an annual COVID-19 booster vaccine (*n* = 919)

	**Total *n (%)***	**Annual COVID-19 booster vaccine**		*P*-value*
		**Acceptant**	**Hesitant**	
***Emotional exhaustion***				
High	273 (29.7%)	85 (31.1%)	188 (68.9%)	.137
Moderate	273 (29.7%)	84 (30.8%)	189 (69.2%)	
Low	373 (40.6%)	139 (37.3%)	234 (62.7%)	
* Total score*	24.6 ± 12.6	23.9 ± 12.8	25.0 ± 12.5	.250
***Depersonalization***				
High	454 (49.4%)	91 (30.3%)	209 (69.7%)	.363
Moderate	193 (21.0%)	82 (35.2%)	151 (64.8%)	
Low	272 (29.6%)	135 (35.0%)	251 (65.0%)	
* Total score*	8.4 ± 6.8	8.1 ± 6.5	8.5 ± 6.9	.529
***Personal accomplishment***				
High	202 (22.0%)	73 (36.1%)	129 (63.9%)	.667
Moderate	145 (15.8%)	48 (33.1%)	97 (66.9%)	
Low	572 (62.2%)	187 (32.7%)	385 (67.3%)	
* Total score*	30.2 ± 11.8	30.6 ± 12.2	30.1 ± 11.6	.341
***Very high Burnout***^**a**^				
Yes	101 (11.0%)	28 (27.7%)	73 (72.3%)	.191
No	818 (89.0%)	280 (34.2%)	538 (65.8%)	

^*^*Chi-squared test and *independent t-test*

^a^HCWs who are categorized high in the three dimensions

##### Determinants of annual COVID booster vaccine acceptance

A logistic regression was performed to ascertain the effects of sex, professional work division, COVID and Influenza vaccination history, burnout construct, and attitudes towards vaccination on the likelihood of accepting an annual COVID booster vaccine. The model explained 40.0% (Nagelkerke R square) of the variance in accepting an annual COVID booster vaccine. HCWs who are suspicious of vaccine benefits [*p*-value < 0.001, aOR = 0.70; 95%CI: 0.65-0.75] and those concerned about unforeseeable future effects [*p*-value 0.001, aOR = 0.90; 95%CI: 0.84-0.95] are less likely to accept an annual COVID-19 booster vaccine if recommended, whereas those who receive annual influenza vaccine are more likely to accept it [*p*-value < 0.001, aOR = 2.9; 95%CI: 1.7–5.0] (Table 4).

**Table 4:** Multivariable analysis of variables associated with willingness to get an annual COVID booster vaccine

	**SE**	*P* Value*	**Adjusted OR (95%CI)**
***Sex***			
Male	.209	.929	1.1 (.68–1.5)
Female^a^			1
***Profession***			
Physicians	.286	.096	1.6 (.92–2.8)
Nurse	.313	.601	1.2 (.64–2.2)
Allied health professionals^a^			1
***Work division***			
Hospitals	.393	.285	1.5 (.71–3.3)
Primary health care	.417	.650	1.2 (.54–2.8)
Other health care settings^a^			1
***COVID-19 vaccination***			
Yes	.244	.208	1.4 (.84–2.2)
No^a^			1
***Received annual influenza vaccine***			
Yes	.275	** < .001**	2.9 (1.7–5.0)
No^a^			1
***Emotional exhaustion***			
High	.290	.652	1.2 (.65–2.1)
Moderate	.288	.949	1.1 (.58–1.8)
Low^a^			
***Depersonalization***			
High	.265	.367	1.3 (76- 2.2)
Moderate	.265	.821	1.1 (.63–1.8)
Low^a^			
***Personal accomplishment***			
High	.245	.144	1.4 (.89–2.3)
Moderate	.282	.941	.59–1.8
Low^a^			1
***Mistrust of vaccine benefits***	.036	** < .001**	.70 (.65-.75)
***Worries over unforeseen future effects***	.033	**.001**	.90 (.84-.95)

*OR* Odds Ratio, *CI* Confidence interval

^a^Reference group

## Discussion

The study reveals that HCWs are generally hesitant to receive an annual COVID-19 booster vaccine, with only one-third accepting it. These findings are significantly lower than those reported among HCWs in high-income countries. For example, in recent surveys, 76.5% of British HCWs [17] and 74.5% of Polish HCWs were willing to receive COVID-19 vaccine booster doses. Higher rates of COVID-19 vaccine booster dose acceptance were reported among HCWs in the United States (83.6%) [31] and China (90.3%) [32]. This subject has not been widely researched in Arab countries. A survey conducted in Saudi Arabia revealed that 67.3% of HCWs expressed willingness to receive the COVID-19 booster vaccine [33].

Generally, the acceptance rates of the COVID-19 vaccine in Arab countries are low and exhibit variability across different countries. A multinational study found that more than half of Arabs are unwilling to receive the COVID-19 vaccine [34]. According to a recent systematic review and meta-analysis, the acceptance rates range from 20% to 97.8%, with countries such as Egypt, Palestine, Jordan, and Oman demonstrating low rates [35]. This limited acceptance may impede the effectiveness of disease control efforts and shape perceptions regarding vaccinations, mainly if the requirement for annual vaccinations is enforced.

The effectiveness of vaccines, particularly the booster vaccine, was a significant factor in the general public's acceptance of the COVID-19 booster vaccine among Algerians and Americans [36, 37]. However, employee organization trust plays a significant role in hesitancy in the UK. It has also been reported that ethnic diversity affects the hesitancy levels of HCWs [17]. In Palestine, and with comparable results in Africa, hesitance to receive a booster vaccination was strongly correlated with a lack of confidence in the value of vaccination [38]. Worries about unforeseen future effects were another factor that hindered the acceptance of COVID-19 booster vaccinations. Also, study participants from Poland and Jordan disagreed that a booster dose of the COVID-19 vaccine would be as safe as the initial doses [39, 40]. Reassuring HCWs of the efficacy of COVID-19 vaccines and being transparent about their side effects are crucial strategies for addressing vaccine benefits and fear of side effects, thereby increasing acceptance of booster vaccination. The publication of new studies showing the vaccine's long-term safety can dispel many HCWs’ concerns and increase the vaccine's acceptance.

Acceptance of an annual COVID-19 booster vaccine is significantly associated with annual influenza vaccination. The logistic regression results indicated that healthcare workers who receive the yearly influenza vaccine are 2.9 times more likely to accept an annual COVID-19 booster vaccine. A review of COVID-19 vaccination hesitancy among HCWs found that previous vaccination habits, particularly for influenza, were associated with support for COVID-19 vaccination [41]. As participation in healthy behavior is expected to be generic, it is assumed that receipt of a previous COVID-19 vaccine should be associated with acceptance of an annual vaccine [31].

The results showed no association between gender and willingness to get the annual COVID-19 booster vaccine, contrary to previous studies. Women have historically been more reluctant to receive vaccinations than men [42], specifically for the COVID-19 vaccine; people surveyed believed that the vaccine could cause infertility [43]. Previous COVID-19 intention and uptake studies demonstrated that female HCWs were less likely to intend to take and certainly take the COVID-19 vaccine [15, 28]. The disappearance of the gender gap for the annual booster COVID-19 in this study could be attributed to the general low acceptance rate among all and the high level of concern about unanticipated future effects shared by both genders. These results emphasize that interventions aimed at increasing the uptake of a booster vaccination amongst Palestinian HCWs should not necessarily be targeted toward either gender.

We did not find a significant association between the HCW profession and acceptance of an annual COVID-19 booster. French nurses were more reluctant to accept the COVID-19 vaccine during the initial pandemic wave than physicians [44]. Similarly, a systematic review study investigated the predictors of vaccine hesitancy and acceptance across different groups and reached an identical conclusion [45]. Although our analysis did not uncover this correlation, the rate of vaccination acceptance among nurses remains low, which may negatively impact the vaccination compliance of individuals who interact professionally or personally with vaccine-hesitant nurses in the future.

The lack of belief in the vaccine's effectiveness and concerns about potential unseen side effects have a negative impact on the willingness to accept an annual COVID-19 booster dose, as indicated by the logistic regression analysis. Studies have shown that mistrust in vaccine efficacy has remained constant throughout the COVID-19 pandemic. This could be attributed to the limited availability of long-term research on the topic and the rapid introduction of the COVID-19 vaccine despite the disparity between early COVID-19 and post-COVID-19 anti-vaccination attitudes [46].

The relationship between HCWs' vaccination hesitancy and Burnout is a significant concern in the healthcare sector. Few studies suggest that vaccine hesitancy among HCWs' can be linked to increased levels of burnout [47]. Galanis P and colleagues found that Greek nurses who had encountered Burnout were less likely to accept a booster COVID-19 vaccine dose [47, 48]. HCWs' unwillingness to receive vaccinations not only indicates a potential lack of understanding about its benefits but also can be attributed to stress and emotional exhaustion [49]. EE was present in one-third of the sample, and high DP and low PA were present in approximately half of the sample. Our results on the prevalence of Burnout are significantly higher than HCWs in Turkey [50]. The burden of pandemic-related challenges and concerns about personal health and safety may exacerbate Burnout among HCWs. This situation presents a dual challenge, as Burnout affects not only individual well-being but also has implications for the overall quality of patient care. Our bivariate analysis results indicated that those who reported higher burnout levels for the three subscales were more likely to be hesitant to receive an annual booster dose, though this was not statistically significant. A survey conducted in the United States revealed that stress does not affect COVID-19 vaccine hesitancy [51].

Our findings should be interpreted with the following limitations in mind. First, the obtained results may be of limited representativeness due to the non-random sampling technique used to recruit participants for this study. Second, our study is susceptible to self-reporting bias as we relied on HCWs to share their views and practices regarding COVID-19 vaccines. This may not be entirely accurate, as people have a tendency to present themselves positively. Comparing these results to future vaccine uptake would be a helpful analysis. Third, even though the first question in the survey asked HCWs if they agreed to participate, an online survey makes estimating the response rate difficult. This may introduce non-response bias, undermining the study's generalizability. Last, the cross-sectional survey design limits our ability to establish causal relationships, and HCWs' attitudes may change over time. Despite these limitations, the study included a large sample of healthcare workers from various sectors, making it one of the first to address this issue in this population group. The findings should aid in a better understanding of the problem and future research.

## Conclusion

In conclusion, the acceptance of an annual COVID-19 booster vaccine is low among Palestinian HCWs. Mistrust of the annual COVID-19 booster vaccine efficacy and concerns about unforeseen side effects remain significant factors in COVID-19 vaccine hesitancy. We recommend launching educational campaigns to address concerns about vaccine benefits and potential long-term effects, as well as developing tailored communication strategies for specific groups, such as female HCWs, nurses, and PHC center staff, to improve understanding and acceptance of the booster vaccine, which helps to increase trust in the COVID-19 vaccine. Healthcare leadership should strongly endorse the significance of vaccinations, encouraging administrators and department heads to set a positive example. On-site vaccination clinics should be established in healthcare facilities, offering flexible scheduling and incentives. Establish a long-term monitoring system to track vaccination adverse effects and complications and the acceptance of annual COVID-19 booster vaccines by HCWs. Regularly assess and adapt strategies based on changing perceptions and needs. Further research is needed to identify and address specific vaccine concerns and build trust among HCWs.

### Supplementary Information

**Supplementary Material 1.**

**Supplementary Material 2.**

## Data Availability

The datasets supporting the conclusions of this article are included within the article and its additional files.

## References

[1] WHO Coronavirus (COVID-19) Dashboard | WHO Coronavirus (COVID-19) Dashboard With Vaccination Data, https://covid19.who.int/ . Accessed 22 Sept 2022.

[2] State of Palestine COVID - Coronavirus Statistics - Worldometer, https://www.worldometers.info/coronavirus/country/state-of-palestine/ . Accessed 22 Sept 2022.

[3] Moynihan R, Sanders S, Michaleff ZA (2021). Impact of COVID-19 pandemic on utilisation of healthcare services: a systematic review. BMJ Open.

[4] Rahmani K, Shavaleh R, Forouhi M (2022). The effectiveness of COVID-19 vaccines in reducing the incidence, hospitalization, and mortality from COVID-19: a systematic review and meta-analysis. Front Public Health.

[5] Feikin DR, Higdon MM, Abu-Raddad LJ (2021). Duration of effectiveness of vaccines against SARS-CoV-2 Infection and COVID-19 disease: results of a systematic review and meta-regression. SSRN Electron J.

[6] Aleem A, Akbar Samad AB, Slenker AK. Emerging Variants of SARS-CoV-2 And Novel Therapeutics Against Coronavirus (COVID-19). Treasure Island: StatPearls Publishing; 2024. 34033342

[7] Episode #53 - COVID-19: Booster Shots, https://www.who.int/emergencies/diseases/novel-coronavirus-2019/media-resources/science-in-5/episode-53---covid-19-booster-shots?gclid=CjwKCAjw5P2aBhAlEiwAAdY7dEKy1PO2Pv3Uq0OL7GFlBDxjFc9mQ6y8lDqCX8qrO8dBN0C5iS8fQxoCZtMQAvD_BwE . Accessed 31 Oct 2022.

[8] Statement by President Biden on FDA and CDC Authorizing Updated COVID-19 Vaccines - The White House, https://www.whitehouse.gov/briefing-room/statements-releases/2022/09/06/statement-by-president-biden-on-fda-and-cdc-authorizing-updated-covid-19-vaccines/ . Accessed 31 Oct 2022.

[9] Iacobucci G (2021). Covid-19: England is preparing to offer annual booster vaccination, says NHS boss. BMJ.

[10] Lin C, Mullen J, Smith D, et al. Healthcare Providers’ Vaccine Perceptions, Hesitancy, and Recommendation to Patients: A Systematic Review. Vaccines; 9. Epub ahead of print July 2021. 10.3390/vaccines9070713. 10.3390/vaccines9070713 PMC8310254 34358132

[11] Klugar M, Riad A, Mohanan L, et al. COVID-19 Vaccine Booster Hesitancy (VBH) of Healthcare Workers in Czechia: National Cross-Sectional Study. Vaccines. 9. Epub ahead of print Dec 2021. 10.3390/vaccines9121437. 10.3390/vaccines9121437 PMC8705445 34960183

[12] Li M, Luo Y, Watson R (2021). Healthcare workers’ (HCWs) attitudes and related factors towards COVID-19 vaccination: a rapid systematic review. Postgrad Med J. Epub ahead of print.

[13] Luo C, Yang Y, Liu Y (2021). Intention to COVID-19 vaccination and associated factors among health care workers: a systematic review and meta-analysis of cross-sectional studies. Am J Infect Control.

[14] Ackah M, Ameyaw L, Gazali Salifu M (2022). COVID-19 vaccine acceptance among health care workers in Africa: a systematic review and meta-analysis. PLoS ONE.

[15] Maraqa B, Nazzal Z, Rabi R, et al. COVID-19 vaccine hesitancy among health care workers in Palestine: A call for action. Prev Med (Baltim). Epub ahead of print 13 May 2021.10.1016/j.ypmed.2021.106618. 10.1016/j.ypmed.2021.106618 PMC8117476 33992654

[16] Rabi R, Maraqa B, Nazzal Z (2021). Factors affecting nurses’ intention to accept the COVID-19 vaccine: a cross-sectional study. Public Health Nurs.

[17] Veli N, Martin CA, Woolf K (2022). Hesitancy for receiving regular SARS-CoV-2 vaccination in UK healthcare workers: a cross-sectional analysis from the UK-REACH study. BMC Med.

[18] Riggio RE. Introduction to Industrial/Organizational Psychology. 7th ed. Routledge/Taylor and Francis Group, 2018.

[19] Maslach C, Leiter MP (2016). Understanding the burnout experience: recent research and its implications for psychiatry. World Psychiatry.

[20] Ghahramani S, Lankarani KB, Yousefi M (2021). A Systematic review and meta-analysis of burnout among healthcare workers during COVID-19. Front Psych.

[21] De Hert S (2020). Burnout in healthcare workers: prevalence, impact and preventative strategies. Local Reg Anesth.

[22] Hamdan M, Hamra AA (2017). Burnout among workers in emergency departments in palestinian hospitals: prevalence and associated factors. BMC Health Serv Res.

[23] Alshawish E, Nairat E (2020). Burnout and psychological distress among nurses working in primary health care clinics in West Bank-Palestine. Int J Ment Health.

[24] Salyers MP, Bonfils KA, Luther L (2017). The relationship between professional burnout and quality and safety in healthcare: a meta-analysis. J Gen Intern Med.

[25] Solís Arce JS, Warren SS, Meriggi NF (2021). COVID-19 vaccine acceptance and hesitancy in low- and middle-income countries. Nat Med.

[26] Paterson P, Meurice F, Stanberry LR (2016). Vaccine hesitancy and healthcare providers. Vaccine.

[27] Martin LR, Petrie KJ (2017). Understanding the dimensions of anti-vaccination attitudes: the vaccination attitudes examination (VAX) scale. Ann Behav Med.

[28] Alya WA, Maraqa B, Nazzal Z (2022). COVID-19 vaccine uptake and its associated factors among Palestinian healthcare workers: Expectations beaten by reality. Vaccine.

[29] Maslach C, Jackson SE, Leiter MP (1997). Maslach burnout inventory.

[30] Sabbah I, Sabbah H, Sabbah S (2012). Burnout among Lebanese nurses: Psychometric properties of the Maslach Burnout Inventory-Human Services Survey (MBI-HSS). Health (Irvine Calif).

[31] Pal S, Shekhar R, Kottewar S, et al. COVID-19 Vaccine Hesitancy and Attitude toward Booster Doses among US Healthcare Workers. Vaccines; 9. Epub ahead of print November 2021. 10.3390/vaccines9111358. 10.3390/vaccines9111358 PMC8617683 34835289

[32] Wu F, Yuan Y, Deng Z (2022). Acceptance of COVID-19 booster vaccination based on the protection motivation theory: a cross-sectional study in China. J Med Virol.

[33] Vellappally S, Naik S, Alsadon O, et al. Perception of COVID-19 Booster Dose Vaccine among Healthcare Workers in India and Saudi Arabia. International Journal of Environmental Research and Public Health. 19. Epub ahead of print 2022. 10.3390/ijerph19158942. 10.3390/ijerph19158942 PMC9332579 35897309

[34] Abu-Farha R, Mukattash T, Itani R (2021). Willingness of Middle Eastern public to receive COVID-19 vaccines. Saudi Pharm J SPJ Off Publ Saudi Pharm Soc.

[35] Wang Q, Hu S, Du F (2022). Mapping global acceptance and uptake of COVID-19 vaccination: a systematic review and meta-analysis. Commun Med.

[36] Lounis M, Bencherit D, Rais MA (2022). COVID-19 Vaccine Booster Hesitancy (VBH) and its drivers in Algeria: national cross-sectional survey-based study. Vaccines.

[37] Yadete T, Batra K, Netski DM, et al. Assessing Acceptability of COVID-19 Vaccine Booster Dose among Adult Americans: A Cross-Sectional Study. Vaccines. 9. Epub ahead of print December 2021. 10.3390/vaccines9121424. 10.3390/vaccines9121424 PMC8703732 34960170

[38] Wiysonge CS, Alobwede SM, de Marie C, Katoto P (2022). COVID-19 vaccine acceptance and hesitancy among healthcare workers in South Africa. Expert Rev Vaccines.

[39] Dziedzic A, Issa J, Hussain S, et al. COVID-19 vaccine booster hesitancy (VBH) of healthcare professionals and students in Poland: Cross-sectional survey-based study. Front Public Heal; 10. Epub ahead of print 2022. 10.3389/fpubh.2022.938067. 10.3389/fpubh.2022.938067 PMC9359622 35958845

[40] Al-Qerem W, Al Bawab AQ, Hammad A, et al. Willingness of the Jordanian Population to Receive a COVID-19 Booster Dose: A Cross-Sectional Study. Vaccines; 10. Epub ahead of print March 2022. 10.3390/vaccines10030410. 10.3390/vaccines10030410 PMC8950968 35335042

[41] Peterson CJ, Lee B, Nugent K. COVID-19 Vaccination Hesitancy among Healthcare Workers-A Review. Vaccines; 10. Epub ahead of print June 2022. 10.3390/vaccines10060948. 10.3390/vaccines10060948 PMC9227837 35746556

[42] Conis E (2013). A mother’s responsibility: women, medicine, and the rise of contemporary vaccine skepticism in the United States. Bull Hist Med.

[43] Sallam M, Dababseh D, Eid H, et al. High Rates of COVID-19 Vaccine Hesitancy and Its Association with Conspiracy Beliefs: A Study in Jordan and Kuwait among Other Arab Countries. Vaccines. 9. Epub ahead of print 2021. 10.3390/vaccines9010042. 10.3390/vaccines9010042 PMC7826844 33445581

[44] Gagneux-Brunon A, Detoc M, Bruel S, et al. Intention to get vaccinations against COVID-19 in French healthcare workers during the first pandemic wave: a cross sectional survey. J Hosp Infect. Epub ahead of print January 2020. 10.1016/j.jhin.2020.11.020. 10.1016/j.jhin.2020.11.020 PMC7699157 33259883

[45] Yasmin F, Najeeb H, Moeed A, et al. COVID-19 Vaccine Hesitancy in the United States: A Systematic Review. Front Public Heal. 9. Epub ahead of print 2021. 10.3389/fpubh.2021.770985. 10.3389/fpubh.2021.770985 PMC8650625 34888288

[46] Huynh HP. Examining four types of anti-vaccination attitudes prior to and during the COVID-19 pandemic. Curr Psychol. 2022;42:1–8. 10.1007/s12144-022-03660-4 PMC9647753 36406857

[47] Galanis P, Katsiroumpa A, Sourtzi P, et al. Social Support Mediates the Relationship between COVID-19-Related Burnout and Booster Vaccination Willingness among Fully Vaccinated Nurses. Vaccines; 11. Epub ahead of print 2023. 10.3390/vaccines11010046. 10.3390/vaccines11010046 PMC9861285 36679890

[48] Peterson CJ, Lee B, Nugent K. COVID-19 Vaccination Hesitancy among Healthcare Workers—A Review. Vaccines; 10. Epub ahead of print 2022. 10.3390/vaccines10060948. 10.3390/vaccines10060948 PMC9227837 35746556

[49] Globevnik Velikonja V, Verdenik I, Erjavec K, et al. Influence of Psychological Factors on Vaccination Acceptance among Health Care Workers in Slovenia in Three Different Phases of the COVID-19 Pandemic. Vaccines; 10. Epub ahead of print November 2022. 10.3390/vaccines10121983. 10.3390/vaccines10121983 PMC9782158 36560393

[50] Alacacioglu A, Yavuzsen T, Dirioz M (2009). Burnout in nurses and physicians working at an oncology department. Psychooncology.

[51] Elliott TR, Perrin PB, Powers MB, et al. Predictors of Vaccine Hesitancy among Health Care Workers during the COVID-19 Pandemic. Int J Environ Res Public Health. 19. Epub ahead of print 2022. 10.3390/ijerph19127123. 10.3390/ijerph19127123 PMC9222587 35742372

