# Supplementary material for: Healthcare workers' attitudes toward and factors influencing their acceptance of an annual COVID-19 booster vaccine: a cross-sectional study in Palestine
Source: BMC Health Serv Res. 2024 May 14;24:624. doi: 10.1186/s12913-024-11016-w (PMC11092075; doi:10.1186/s12913-024-11016-w)
Supplement: Supplementary file 1 — Supplementary Material 1. [file 12913_2024_11016_MOESM1_ESM.pdf]

Questionnaire

Dear Colleague,

You are invited to participate in a research project being conducted by Dr Zaher Nazzal and his colleagues. Dr Zaher is an associate presser of Community Medicine at An-Najah National University. The purpose of this research is to assess the willingness of Palestinian healthcare professionals to receive an annual COVID-19 booster vaccination, if it is recommended. If you decide to participate, you will be asked to complete an anonymous web-based survey that should take no more than 10 minutes. The survey will not collect any identifiable information and no one will be able to connect your responses to you. Your anonymity is further protected by not asking you to sign and return a consent form. Your completion of the survey will serve as your consent. If you have any questions about this study, you may call me at +972599545421or my colleague, Dr. Beesan, +972 59-938-7195. This project has been reviewed and approved by An-Najah National University Review Board.

Thank you for your time.

**Section I: Background and clinical**				
**Residency (district)**	**…………………….**	**Work place (district) …………………….**		
**Gender**	☐ Male	☐ Female	**Age (year) ………………..**	
**Marital status**	☐ Married	☐ Single	☐ Divorced/ Widowed	
**Job title**	☐ Physician<br>☐ Nurse	☐ Paramedics *(lab technicians, radiology, and occupational and physiotherapists, etc)*<br>☐ Others…………………..		
**Work setting**	☐ Primary health care<br>☐ Governmental hospital	☐ Private hospital<br>☐ NGOs		
**Smoking history** (You can choose more than one answer)				
	☐ Yes<br>☐ I used to, but I quitted before the pandemic.	☐ No<br>☐ I used to, but I quitted during the pandemic.		
**Do you have chronic diseases?** (You can choose more than one answer)				
	☐ No	☐ HTN	☐ DM	☐ IHD
	☐ Chronic respiratory disease	☐ Cancer	☐ CKD	☐ Others<br>Specify: ……..…….
**Work experience (total years): ………………………….**				

**Section II: COVID-19 and vaccination**			
Did you get infected with CORONA Virus previously (PCR+ve)?		☐ Yes	☐ No
Have you dealt with a patient who developed severe symptoms after receiving the COVID-19 vaccine?		☐ Yes	☐ No
Have you received at least two doses of coronavirus vaccines?		☐ Yes	☐ No
If **yes**	Did you experience side effects following vaccination?	☐ No<br>☐ Yes, Mild Moderate<br>☐ Yes, Mild Moderate	☐ Yes, severe<br>☐ Yes, very severe
Do you receive the influenza virus vaccine annually?		☐ Yes	☐ No

Would agree to receive an annual COVID-19 booster vaccine if it is recommended?	☐ Yes<br>☐ No<br>☐ Not decided yet

Questionnaire

Section III: Attitude to vaccination					
**Please mark the choices that most accurately reflect your feelings or beliefs**					
	**Strongly agree**	**Agree**	**Neutral**	**Disagree**	**Strongly disagree**
I feel safe after being vaccinated with the COVID-19 Vaccine.					
I can rely on the COVID 19 vaccines to stop the COVID-19 disease.					
Natural exposure to viruses and germs gives the safest protection.					
Although most vaccines appear to be safe, there may be problems that we haven't yet discovered					
The Vaccine can cause unforeseen problems.					
Authorities promote vaccination for financial gain, not for people's health.					
Being exposed to diseases naturally is safer for the immune system than being exposed through vaccination.					
I worry about the unknown effects of the Vaccine in the future					
The Vaccination program is a big con					
Natural immunity lasts longer than vaccination.					
I feel protected after getting vaccinated					
The Vaccine makes a lot of money for pharmaceutical companies but does not do much for regular people.					

Section IV: Burnout							
**Please mark the choices that most accurately reflect your feelings**	**0** = *Never*<br>**1** = *At least a few times a year*<br>**2** = *At least once a month*			**3** = *Several times a month*<br>**4** = *Once a week*<br>**5** = *Several times a week*<br>**6** = *Every day*			
	**0**	**1**	**2**	**3**	**4**	**5**	**6**
I feel emotionally exhausted because of my work							
I feel worn out at the end of a working day							
I feel tired as soon as I get up in the morning and see a new working day stretched out in front of me							
I can easily understand the actions of my colleagues/supervisors							
I get the feeling that I treat some clients/colleagues impersonally, as if they were objects							
Working with people the whole day is stressful for me							
I deal with other people's problems successfully							
I feel burned out because of my work							
I feel that I influence other people positively through my work							
I have become more callous to people since I have started doing this job							
I'm afraid that my work makes me emotionally harder							
I feel full of energy							
I feel frustrated by my work							
I get the feeling that I work too hard							
I'm not really interested in what is going on with many of my colleagues							
Being in direct contact with people at work is too stressful							
I find it easy to build a relaxed atmosphere in my working environment							
I feel stimulated when I been working closely with my colleagues							
I have achieved many rewarding objectives in my work							
I feel as if I'm at my wits 'end							
In my work I am very relaxed when dealing with emotional problems							
I have the feeling that my colleagues blame me for some of their problems							

Thank You
